# Genome analysis of orf virus isolates from goats in the Fujian Province of southern China

**DOI:** 10.3389/fmicb.2015.01135

**Published:** 2015-10-23

**Authors:** Xuelin Chi, Xiancheng Zeng, Wei Li, Wenbo Hao, Ming Li, Xiaohong Huang, Yifan Huang, Daniel L. Rock, Shuhong Luo, Shihua Wang

**Affiliations:** ^1^College of Life Sciences, Fujian Agriculture and Forestry UniversityFuzhou, China; ^2^University Key Laboratory for Integrated Chinese Traditional and Western Veterinary Medicine and Animal Healthcare in Fujian Province, College of Animal Sciences, Fujian Agriculture and Forestry UniversityFuzhou, China; ^3^Department of Immunology, School of Biotechnology, Southern Medical UniversityGuangzhou, China; ^4^Department of Pathobiology, College of Veterinary Medicine, University of Illinois at Champaign-UrbanaUrbana, IL, USA

**Keywords:** *Parapoxvirus*, orf virus, gene deletion, gene variation, phylogenetic tree

## Abstract

Orf virus (ORFV), a species of the genus *Parapoxvirus* of the family *Poxviridae*, causes non-systemic, highly contagious, and eruptive disease in sheep, goat, and other wild and domestic ruminants. Our previous work shows orf to be ubiquitous in the Fujian Province of China, a region where there is considerable heterogeneity among ORFVs. In this study, we sequenced full genomes of four Fujian goat ORFV strains (OV-GO, OV-YX, OV-NP, and OV-SJ1). The four strains were 132–139 kb in length, with each containing 124–132 genes and about 64% G+C content. The most notable differences between the four strains were found near the genome termini. OV-NP lacked seven and OV-SJ1 lacked three genes near the right terminus when compared against other ORFVs. We also investigated the skin-virulence of the four Fujian ORFVs in goats. The ORFVs with gene deletions showed low virulence while the ORFVs without gene deletions showed high virulence in goats suggesting gene deletion possibly leads to attenuation of ORFVs. Gene 134 was disrupted in OV-NP genome due to the lack of initial code. The phylogenetic tree based on complete *Parapoxviruse* genomes showed that sheep originated and goat originated ORFVs formed distinctly separate branches with 100% bootstrap. Based on the single gene phylogenetic tree of 132 genes of ORFVs, 47 genes can be easily distinguished as having originated from sheep or goats. In order to further reveal genetic variation presented in goat ORFVs and sheep ORFVs, we analyzed the deduced amino acid sequences of gene 008, multiple alignment of amino acid sequences of gene 008 from the genome of five goat ORFVs and four sheep ORFVs revealed 33 unique amino acids differentiating it as having sheep or goats as host. The availability of genomic sequences of four Fujian goat ORFVs aids in our understanding of the diversity of orf virus isolates in this region and can assist in distinguishing between orf strains that originate in sheep and goats.

## Introduction

Orf, known as contagious ecthyma, is a non-systemic cutaneous and debilitating disease with worldwide distribution. Sheep and goats are the most easily infected. In addition, camels, deer, reindeer, musk ox, Japanese serows can also become infected (Kummeneje and Krogsrud, 1978, 1979; Inoshima et al., 2002). Clinically, the lesions of the disease tend to proliferate on the mouth and oral mucosa as well as around the nostrils. Lesions follow a characteristic development pattern: erythema followed by the formation of papules, vesicles, pustules, and scabs. The disease also has zoonotic potential. Humans, especially those working with animals (veterinarians, farmers, butchers), can be infected by orf (Kumar et al., 2014). In humans, the most common lesions are self-limiting with painful pustules on the hands, face, and arms (Mazur et al., 2000; Al-Salam et al., 2008).

*Parapoxviruses* (PPV) are classified as a genus within the *Poxviridae* family, which are large, enveloped, double-stranded DNA viruses. PPV includes four species, *Bovine popular stomatitis virus* (BPSV), *Pseudocowpoxvirus* (PCPV), *Orf virus* (ORFV) and a newly defined virus found in a species of red deer in New Zealand (PVNZ) (Friederichs et al., 2014). ORFV is the prototype member of PPV. By contrast with other poxviruses, ORFV is one of the most extensively studied viruses due to its worldwide distribution and its zoonotic potential. At present, there are eight complete genomes of PPVs available in Genbank, including five ORFV strains, one BPSV strain, and two PCPV strains (Table 1). The five ORFVs are IA82, NZ2, D1701, NA1/11 and SA00, and, among of these strains, only SA00 originated from a goat while the other strains originated from sheep. With the exception of D1701, these PPVs predicted 131–134 genes, retaining the numbering of Delhon et al. (2004).

**Table 1:** **Summary of genomic sequence data of 12 PPV strains**.

**PPV species**	**Isolate**	**Host**	**No. predicted genes**	**Genome size bp**	**ITR size bp**	**Genome G+C %**	**Genbank accession. no.**	**References**
ORFV	GO	Goat	132	139886	3964	63.6	KP010354	In this study
ORFV	YX	Goat	132	138231	3446	63.8	KP010353	In this study
ORFV	SJ1	Goat	129	139112	4153	63.6	KP010356	In this study
ORFV	NP	Goat	124	132111	2426	63.8	KP010355	In this study
ORFV	SA00	Goat	132	139962	3936	63.4	AY386264	Delhon et al., 2004
ORFV	IA82	Sheep	132	137241	3092	64.3	AY386263	Delhon et al., 2004
ORFV	NZ2	Sheep	132	137820	3389	64.3	DQ184476	Mercer et al., 2006
ORFV	NA1/11	Sheep	132	137080	3020	63.6	KF234407	Li et al., 2014
ORFV	D1701	Sheep	288	134038			HM133903	Cottone et al., 1998; McGuire et al., 2012
PCPV	VR634	Human	134	145289	14909	65.0	GQ329670	Hautaniemi et al., 2010
PCPV	F00.120R	Reindeer	131	134600	2800	64.1	GQ329669	Hautaniemi et al., 2010
BPSV	AR02	Calf	133	134431	1161	64.5	AY386265	Delhon et al., 2004

In recent years, there have been several orf outbreaks reported within China. The phylogenetic analysis of ORFVs based on single genes was used to evaluate its molecular epidemiology and distribution characteristics (Li et al., 2012; Yang et al., 2014). However, the entire genomic sequence of the ORFV from goats in China is not currently available. In our previous study, eight ORFV strains were successfully isolated from goats in different areas within the Fujian Province (China) and phylogenetic analysis based on ORFV011 and ORF059 genes revealed that these isolates are highly divergent. In addition, our previous research showed the severity of symptoms caused by ORFVs may be associated with the type of genome (Chi et al., 2013). To better understand the relationship of ORFV virulence and disease pathogenesis with genome sequence variation among different isolates, we determined the genomic sequences of four typical ORFV strains (OV-GO, OV-SJ1, OV-NP, and OV-YX) from goats in Fujian Province, using the phylogenetic results of ORFV011 and ORFV059 as our basis. The availability of genomic sequences of four Fujian goat ORFVs aids in our understanding of the diversity of ORFV isolates in this region and enabled us, for the first time, to compare ORFV strains with either goat or sheep origination at the genome level, which provides a basis for the development of specific orf vaccines.

## Materials and methods

All animal protocols used in this study have been revised and approved by Fujian Agriculture and Forestry University Animal Care and Use Committee (Certification Number: CNFJAC0027).

### Virus strains

ORFV strain GO, YX, SJ1, NP were isolated in Fujian Province, southern China from scab materials collected from goats with multifocal, proliferative dermatitis (Chi et al., 2013). Other PPV genomes used in this analysis, obtained from Genbank, are listed in Table 1. These include four ORFVs isolated from sheep, one ORFV strain isolated from goats, two PCPVs, and one BPSV.

### DNA sequencing, assembly, and annotation

OV-YX, OV-GO, OV-SJ1, and OV-NP were cultured but they had not been passaged more than six times and purified as previously described (Chi et al., 2013). Viral DNA was prepared using the QIAamp DNA blood kit (QIAGEN, Germany) according to the manufacturer's instructions. The genome of ORFV field strain YX, GO, SJ1, and NP were sequenced using an Illumina Hiseq2000 at BGI (BGI Shenzhen, China) (Zeng et al., 2014). Contigs were assembled and scaffolds were constructed using clean data with SOAP denovo 2.04. ZOOM studio 1.5 (Bioinformatics Solutions Inc.) has been used to map millions of original short reads, confirming the four ORFV genome were assembled at high coverage (200–450 fold coverage) and with good accuracy. The sequence data was further analyzed using BioEdit (Ibis Bioscience, CA) to identify gaps between scaffolds. Gaps were closed as described previously (Afonso et al., 1999). Open reading frames (ORFs) were identified with the NCBI ORF Finder (http://www.ncbi.nlm.nih.gov/gorf/gorf.html) referring to other ORFV genomes.

### Sequence alignment and phylogenetic analysis

Phylogenetic analyses were performed on the all genomic nucleotide sequences of PPVs including terminal repetition (12 strains in total, listed in Table 1) and each gene sequences (132 predicted genes in total) of 11 PPVs with the MEGA 5.0 software (Tamura et al., 2011; Hall, 2013). Single gene sequences of D1701 were not aligned because gene numbering was inconsistent with other PPVs. Individual nucleotide sequences and complete genome sequences were aligned by using ClustalW (Thompson et al., 1994) with choosing Align Codons and Align DNA respectively, and the alignments concatenated into 133 separate blocks of data. The bootstrap method was chosen to estimate the reliability of a phylogenetic tree. For genome sequences, the Tamura-Nei model was determined with the default setting. For each gene individually, model test was used to identify the best evolution model. When completed the models were listed in a table in order of preference. Then the tree was estimated using the most appropriate model. There were 77 genes (001, 009, 010, 011, 015, 016, 017, 018, 019, 020, 022, 023, 024, 025, 026, 027, 029, 032, 034, 035, 036, 037, 038, 039, 040, 045, 047, 049, 050, 051, 052, 053, 054, 055, 057, 058, 062, 064, 067, 070, 071, 072, 073, 074, 076, 079, 080, 081, 082, 084, 087, 089, 090, 093, 094, 095, 096, 097, 098, 100, 105, 106, 107.5, 108, 113, 114, 120, 122, 123, 124, 126, 128, 129, 130, 131, 132, and 134) with the T92 (Tamura 3-parameter)+G (a gamma distribution of rate variation) model, and 13 genes (007, 013, 014, 041, 043, 046, 048, 063, 085, 088, 111, 117, and 125) with the T92+I (a proportion of invariable sites) model, and nine genes (056, 060, 068, 069, 075, 083, 086, 103, and 112) with the GTR (General Time Reversible)+G model, and seven genes (008, 028, 044, 059, 065, 066, and 078) with T92+G+I model, and seven genes (002, 031, 077, 092, 099, 118, and 119) with the T92 model, and five genes (012.5, 021, 030, 061, and 101) with the HKY (Hasegawa-Kishino-Yano)+G model, and five genes (005, 102, 104, 110, and 115) with the K2 (Kimura 2-parameter)+G model, and two genes (091 and 107) with the JC (Jukes-Cantor) model, and two genes (012 and 042) with the JC+G mode, and gene 033 with the TN93 (Tamura-Nei)+I model, gene 109 with the GTR+I model, gene 116 with the HKY model, gene 121 with the HKY+I model, and gene 127 with the K2+I model. For all concatenated alignments, maximum-likelihood trees were constructed with 1000 bootstrap replicates.

### Nucleotide sequence accession number

The four ORFV genome sequences were submitted to GenBank with the following Accession Numbers: OV-YX: KP010353; OV-GO: KP010354; OV-NP: KP010355; OV-SJ1: KP010356.

### PCR confirmed the deleted region in the right terminal of OV-NP and OV-SJ1 genome and the disrupted gene 134 in OV-NP genome

In order to confirm the reality of deletion, a primer pair based on the two ends of deletion region of OV-NP genome was to amplify a 1290 bp region spanning the deletion in OV-NP genome and similarly, a primer pair was to amplify a 754 bp region spanning the deletion in OV-SJ1 genome. In all ORFV genomes except OV-NP, gene 134 completely locates at the ITR region while only part of gene 134 locates at the ITR region with disruption of this gene. To confirm the disruption of gene 134 in OV-NP genome, a primer was to amplify a 1229 bp region spanning the node of the beginning of ITR at the right end of OV-NP genome. The primer sequences were as follows:

NP113-121F-1290 bp (forward primer): 5′-TGGG AAATGGATCTGTCGA-3′;
NP113-121R-1290 bp (reverse primer): 5′-CAGT TGGAAGAGTGGTGGTGT-3′;
SJ1117-119F-754 bp (forward primer): 5′-CAC TCGGCGCAATGGATC-3′;
SJ1117-119F-754 bp (reverse primer): 5′-GCT GTCGTTGAGCGTCTCG-3′;
NP-134-gene-F (1229 bp) (forward primer): 5′-TGC GAATGTATTGATGGAA-3′;
NP-134-gene-R (1229 bp) (reverse primer): 5′-TCT GAGTGAGGAGCGAGTT-3′.

### Animal inoculation

In an independent experiment, five groups were set up to investigate the ORFV virulence of OV-YX, OV-SJ1, OV-GO, and OV-NP in goats. The five groups in the experiment consisted of mock-infected (*n* = 2), OV-YX-infected (*n* = 2), OV-GO-infected, OV-SJ1-infected (*n* = 2) and OV-NP-infected (*n* = 2) kids. Three-to-six-month-old crossbred kids with serum orf antibody negative by ELISA were equally distributed among the five groups. Each kid was inoculated in the mucocutaneous junction of the inferior lips and the inner side of their thighs. Kids were tranquilized with phenobarbital (Luminal; Bayer), and the inner side of their thighs were sheared. Then the sites of inoculation were cleaned with physiological saline. After the scarification of inoculation sites, 0.5 ml of virus suspension containing 10^5.4^ TCID_50_ was inoculated at each site by using a cotton swab. Animals were monitored for 1 month for characteristic orf lesions.

## Results

### Genomic features of the four goat ORFV strains from fujian province, China

**Figure 1:**
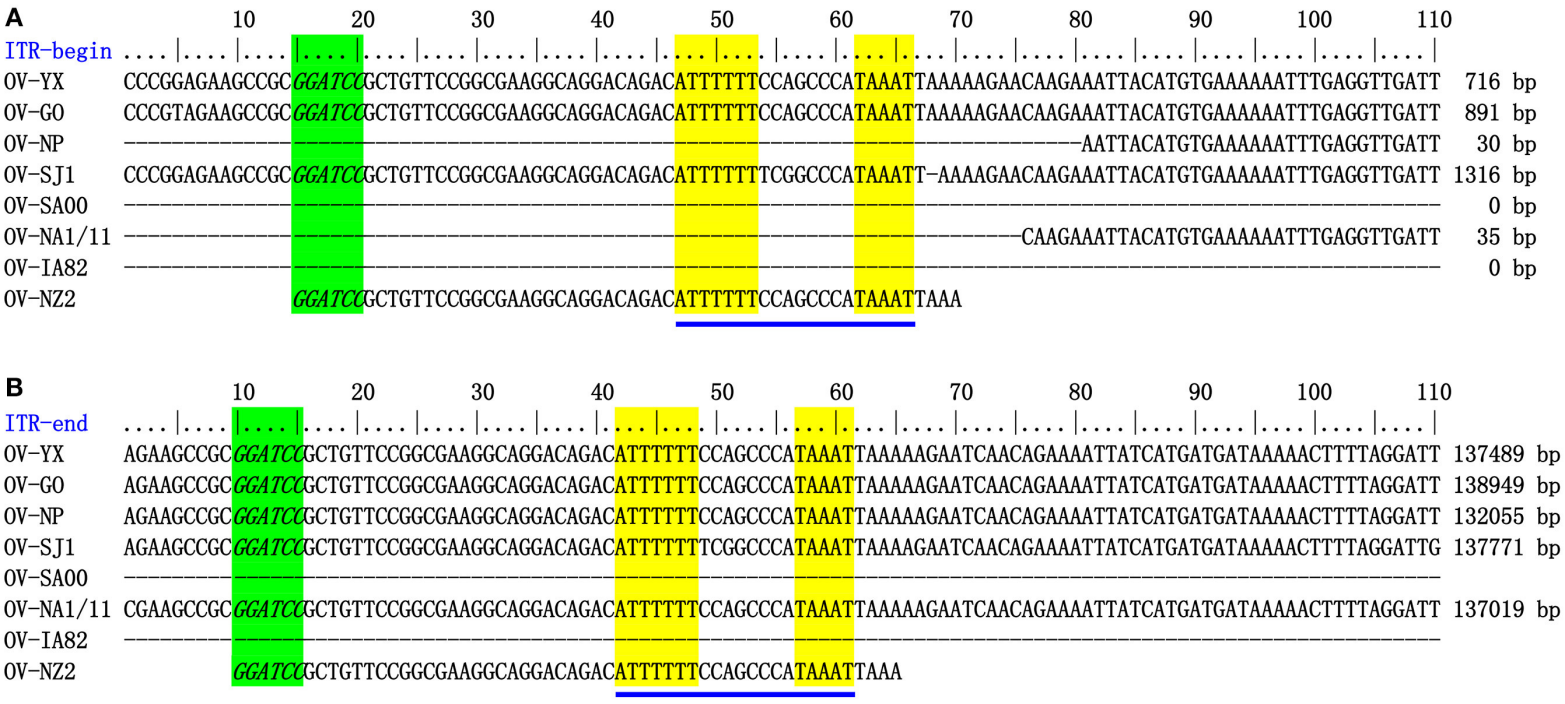
**ITR-ends. (A)** Sequence external to the terminal BamHI site (italicized, green) is aligned with the left end terminal sequence of eight ORFV genomes. **(B)** With the right end terminal sequence of eight ORFV genomes. The telomere resolution motifs were underlined in yellow (ATTTTTT-N(8)-TAAAT).

The genome sequences of ORFV field isolate strains GO, SJ1, YX, NP were assembled into contiguous sequences of 139,886, 139,112, 138,231, 132,111 bp (Table 1). Like other poxviruses, the terminal regions of these four ORFV genomes contained inverted terminal repeats (ITRs). The ITRs of GO, SJ1, YX, NP were 3964, 4153, 3446, and 2426 bp, respectively. The sequences of GO, SJ1, and YX contained bases outside of terminal *Bam*HI sites at both ends, but the sequence of NP only contained bases outside of the terminal *Bam*HI sites at the right end. After aligning sequences external to the terminal *Bam*HI sites, YX, SJ1, and GO sequences all contained a conserved motif required for resolution of poxviral concatameric DNA replicative intermediates at both end of the genomes, but the NP sequence only contained this resolution sequence at the right end (Figure 1), suggesting these assemblies except for NP include the terminal hairpin loops. Among other ORFV genome sequences, only NA1/11 contained this resolution sequence at the right end. An additional 49 bp sequence beyond the terminal *Bam*HI site of NZ2 contained this conserved resolution sequence. In ORFV genomes, the right-hand region of the resolution sequence can be represented as TAAAT. The next eight nucleotides, ACCCGACC, functioned as a spacer region as changes within this stretch, followed by six T residues. Figure 1 also shows that the region between *Bam*HI sites to the conserve resolution sequence were completely consistent with each other. Like other PPV genomes, the four genomes possessed approximately 64% G+C content with a distinctive pattern, genus-specific G+C profile typical of PPVs (Hautaniemi et al., 2010).

The ORFs of four ORFV field strain genomes were predicted by using BLASTP comparisons of the predicted amino acid sequence with the GenBank database and poxvirus protein. The coding potentials of the GO, SJ1, YX, NP were predicted to contain 132, 129, 132, 124 genes, respectively (See Supplementary Table S1). Numbering of the genes was adopted from Delhon et al. (2004) and Mercer et al. (2006) in order to allow comparisons with other published PPV genomes. In this consecutive numbering of genes, 003, 004, 006, and 133 were not used in the ORFV genome. Similarities to well-defined proteins in the ORFV NZ2, IA-82, and SA00 reference genomes were found, and the four genomes included two newly recognized genes (12.5 and 107.5) by Mercer et al. (2006). In the GO, YX, and SJ1 genomes, one predicted gene appeared twice completely located within the inverted terminal repeat (001/134). However, in the NP genome, only 178 bp of one predicted gene (447 bp) was located in the inverted terminal repeat. In the NP genome, gene 001 was intact, but the corresponding gene 134 was disrupted due to the lack of initiation coding. This is not consistent with other ORFV genomes. Sanger sequencing confirmed that gene 134 in the NP genome was disrupted and each ITR of NP did not include one predicted gene (data not shown).

### Discovery of genes deletion in NP and SJ1 genome

**Figure 2:**
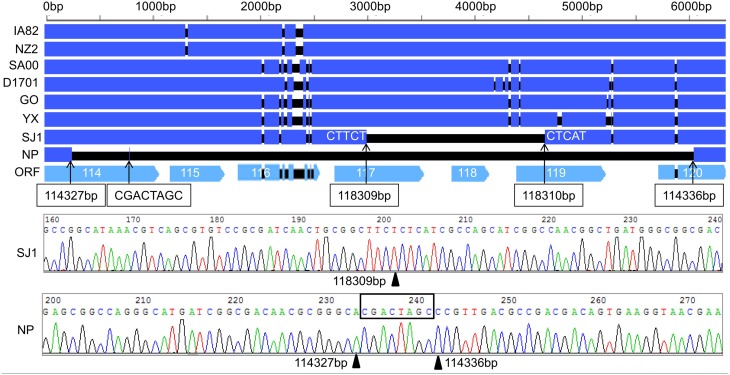
**Alignment of the sequences of gene 114–120 revealed the gene deletion region in OV-NP and OV-SJ1 genomes, and sequences of the fragments spanning the deletion (By Sanger sequencing PCR products) confirming the deletion**.

During analysis of the coding potential of the NP and SJ1 genome, it was surprising to find no sequence corresponding to genes 115–118, the right terminal part of gene 114 and the left terminal part of gene 120 (approximately 5.7 kb in length) in the NP genome, and no corresponding sequence to gene 118, the right terminal part of gene 117, and the left terminal part of gene 119 (approximately 1.5 kb in length) in the SJ1 genome (Figure 2). PCR specific primers based on both ends of the deletion region were designed to further confirm the deletion. Corresponding fragments spanning the deletion were found in the NP and SJ1 genome. Sequencing of the fragments confirmed that the deletions really occurred in the NP and SJ1 genomes (Figure 2).

### Comparison of ORFV virulence in goats

**Figure 3:**
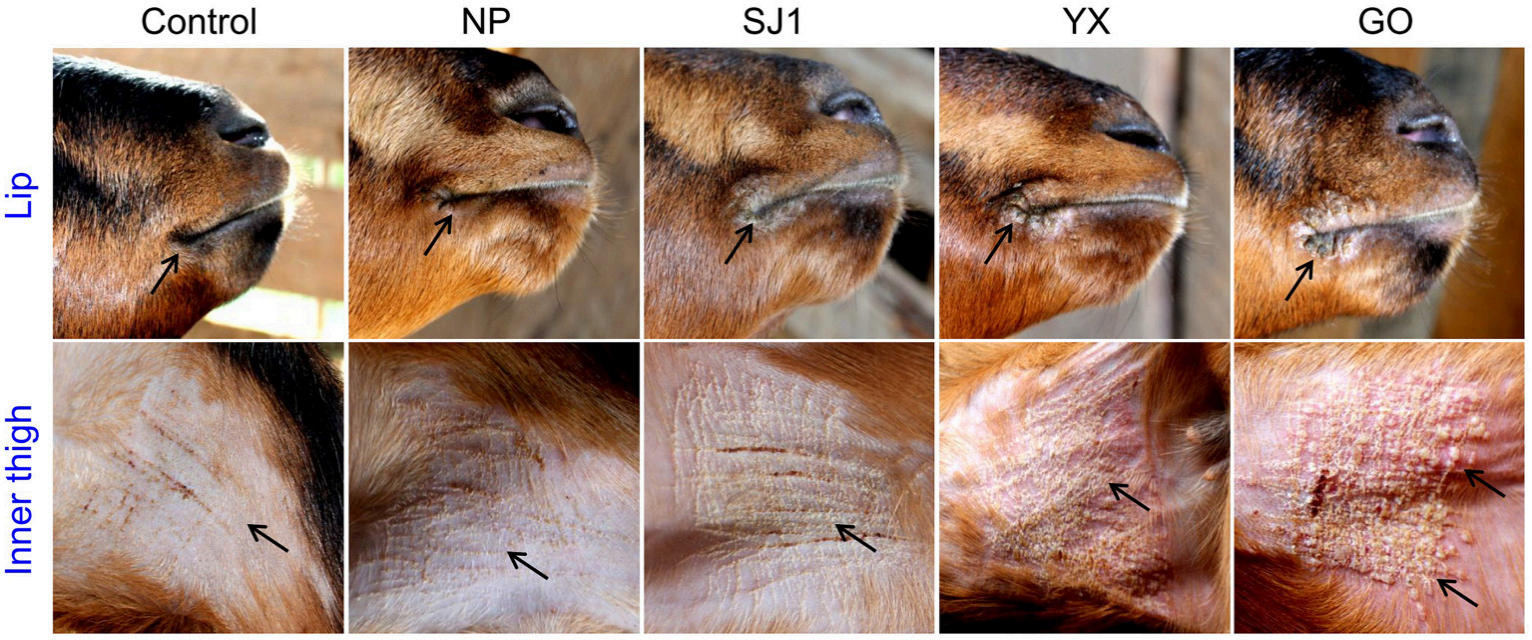
**Skin virulence of four Fujian goat ORFVs in 6 days post-infection**. Clinical features of orf in kids inoculated with OV-NP,OV-SJ1,OV-GO,OV-YX viruses, or MEM (control) at the right mucocutaneus junction of the lips and on the inner thighs.

An animal experiment was set up to investigate the virulence of OV-YX, OV-SJ1, OV-GO, and OV-NP. In this experiment, 3–6 month-old kids were inoculated with OV-NP, OV-SJ1, OV-YX, OV-GO, or MEM (control group) and clinical orf was observed. In OV-YX and OV-GO virus-inoculated kids, lip lesions were observed initially by day 3 p.i., and scabs were observed by day 4 p.i. By day 6 p.i., scabs on the lips were obvious in OV-GO and OV-YX virus-inoculated kids (Figure 3). In OV-YX virus-inoculated kids, lesions began to resolve by day 10 p.i., and by 16 day p.i., no scabs were observed on the lips. In OV-GO virus-inoculated kids, lesions began to resolve by day 16 p.i. By day 20 p.i., there were still small scabs on the lips. In OV-SJ1 virus-inoculated kids, lip lesions were very mild (Figure 3). By day 6 p.i., tiny scabs were observed on the lips, and by day 8 p.i., scabs were completely resolved. Control group animals and OV-NP inoculated animals did not exhibit significant changes (Figure 3). A similar result was observed on the inner side of the thighs. In OV-YX and OV-GO virus-inoculated kids, lesions consisted of erythema, and pustules were first observed by day 4 p.i. At 6 days p.i., pustules were obvious, especially in OV-GO virus–inoculated kids (Figure 3). In OV-YX virus-inoculated kids, scabs on the inner thighs were observed by day 9 p.i., and scabs were completely resolved by day 18 p.i. In OV-GO virus-inoculated kids, scabs on the inner thighs were observed by day 11 p.i., and completely resolved by day 24 p.i. OV-NP and OV-SJ1 virus-inoculated animals exhibited inconspicuous changes, and only minor scabs were observed, and minor scabs began to resolve in the OV-NP virus-inoculated group (Figure 3). Control group animals showed minor scabs induced by skin scarification, and some scabs had been resolved by day 6 p.i. (Figure 3). These results indicated that the virulence of OV-GO is the strongest among the four ORFV strains, the second being OV-YX, while OV-SJ1 and OV-NP exhibited low virulence in goats.

### Comparison of four goat ORFV strains from fujian region, China with other ORFVs

**Figure 4:**
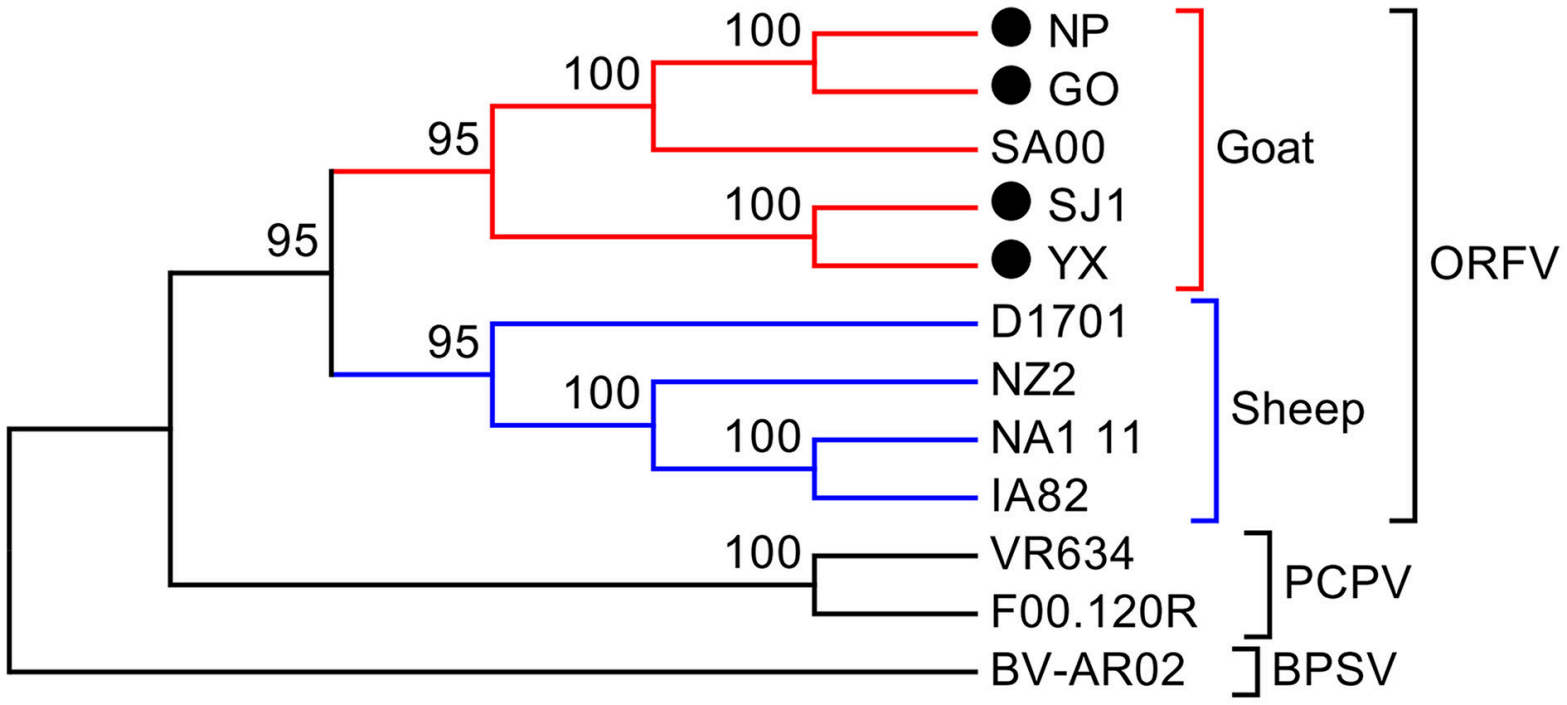
**Phylogenetic comparison of PPVs**. Genomic nucleotide sequences including terminal repetition were aligned using Clustal W. Phylogenetic trees were generated applying the maximum-likelihood algorithm using MEGA 5.0 software. Numbers at the branching points indicate the bootstrap support calculated for 1000 replicates.

**Figure 5:**
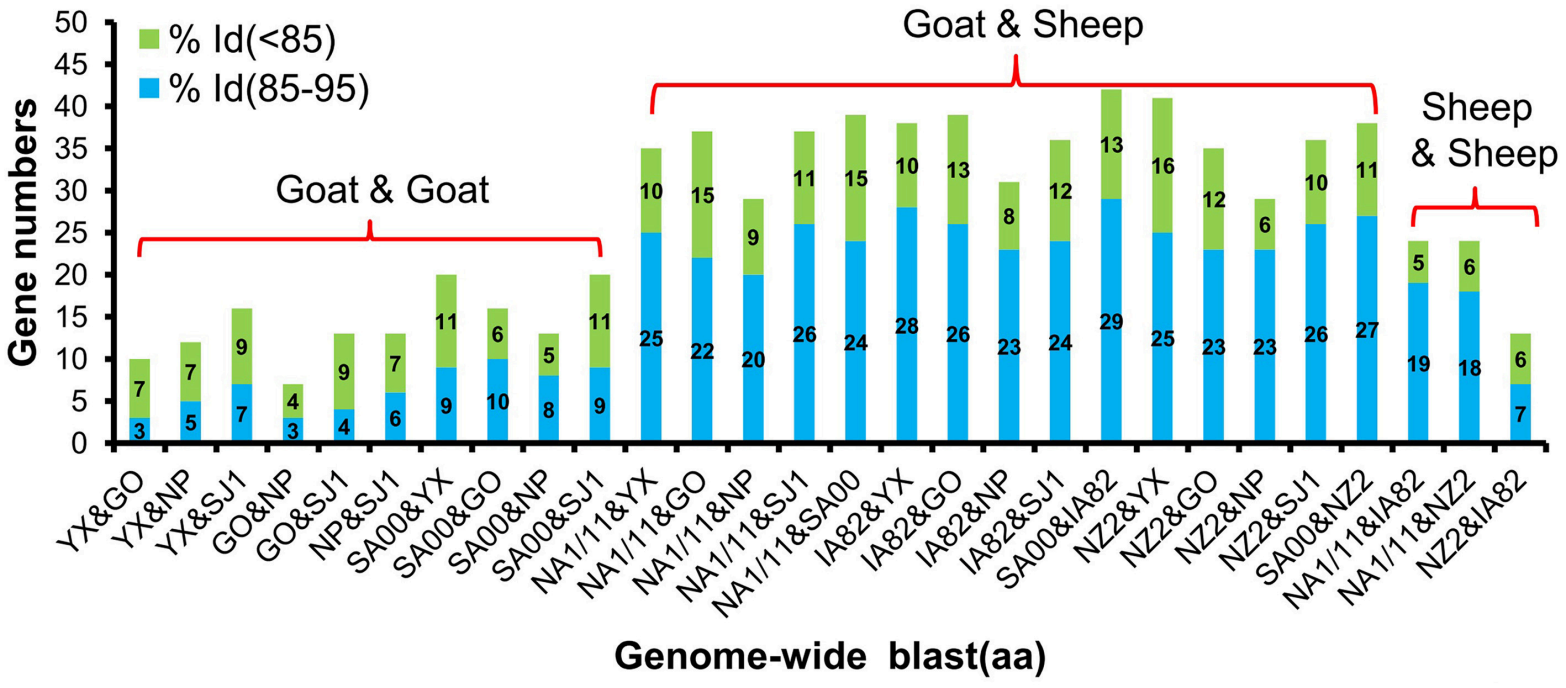
**Gene numbers of below 95% and 85–95% amino acid identity compared with each other among eight ORFVs**.

**Figure 6:**
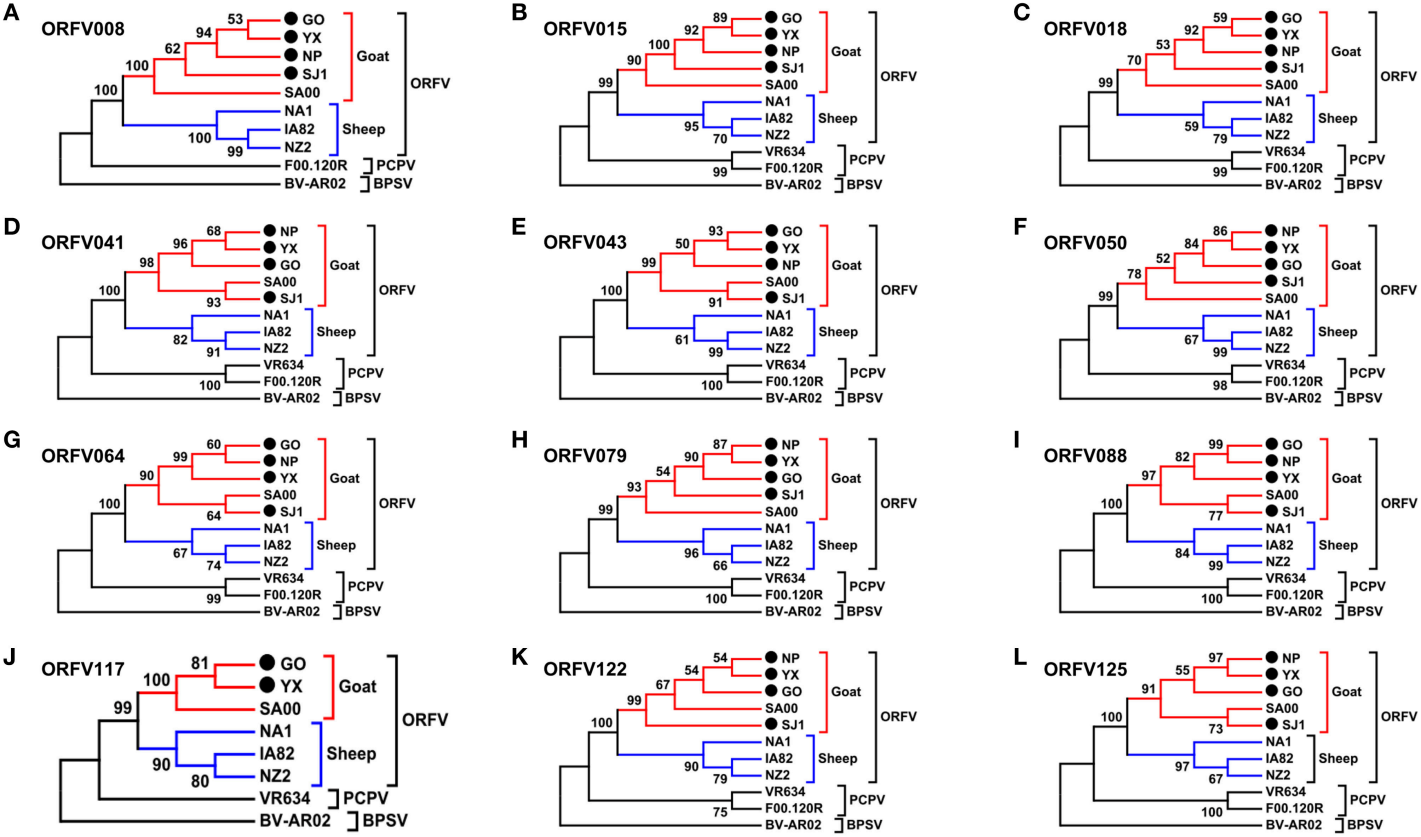
**Phylogenetic analysis based on nucleotide sequence of ORFV single gene distinguishing goat and sheep origination**. The phylogenetic relationship was constructed by the maximum-likelihood method using MEGA 5.0 software. Numbers at the branching points indicate the bootstrap support calculated for 1000 replicates. **(A)** ORFV008. **(B)** ORFV015. **(C)** ORFV018. **(D)** ORFV041. **(E)** ORFV043. **(F)** ORFV050. **(G)** ORFV064. **(H)** ORFV079. **(I)** ORFV088. **(J)** ORFV117. **(K)** ORFV122. **(L)** ORFV125.

**Figure 7:**
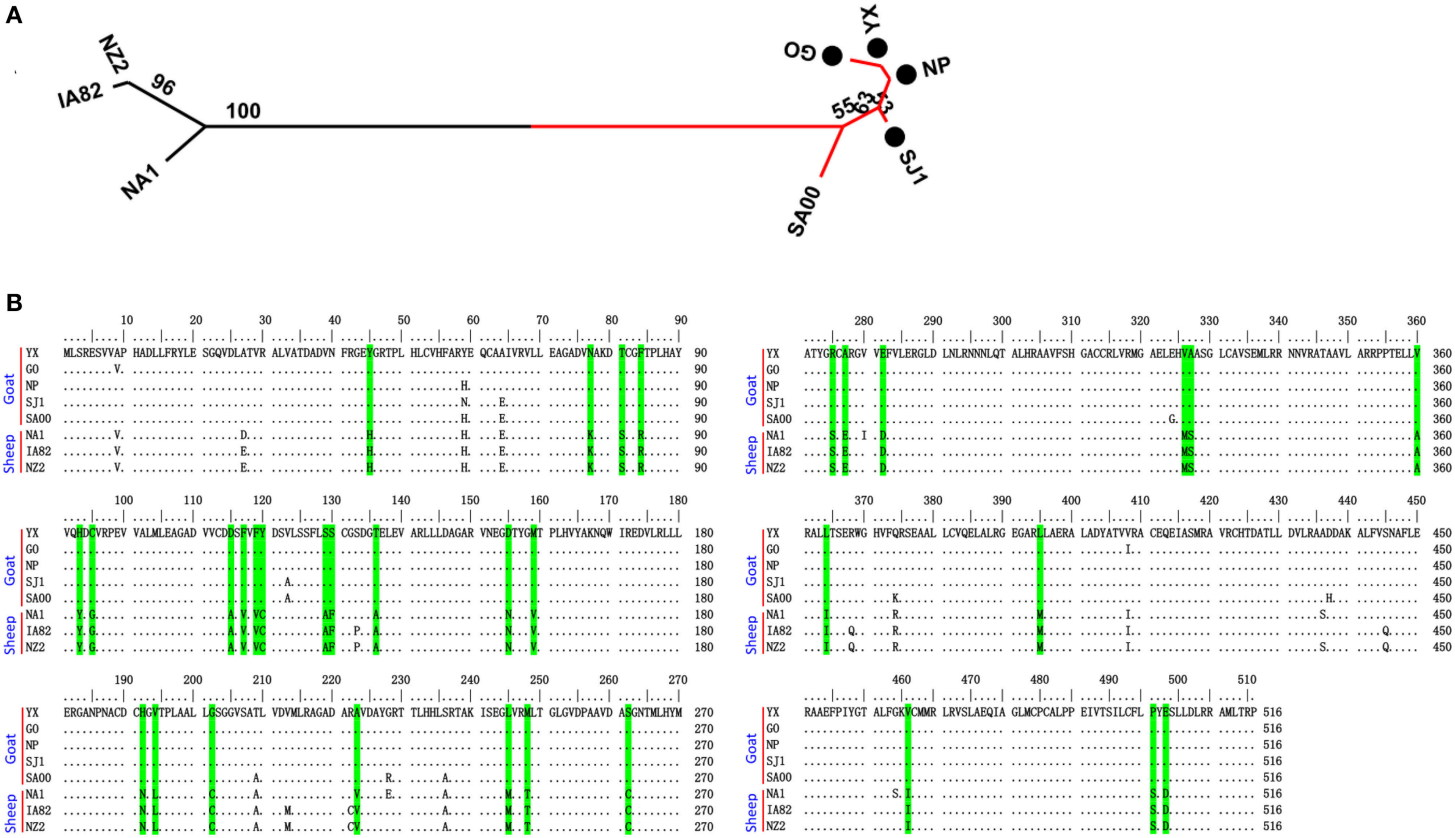
**Phylogenetic analysis and multiple alignment of deduced amino acid sequence of ORFV008. (A)** The phylogenetic relationship was constructed by the maximum-likelihood method using MEGA 5.0 software. Numbers at the branching points indicate the bootstrap support calculated for 1000 replicates. Black cycles: four Fujian goat ORFVs. **(B)** Green marks are unique amino acid residues in goat genomes.

Phylogenetic analyses based on the whole genome showed that ORFVs with goat origination and sheep origination formed separate branches with 100% bootstrap support. The four ORFV strains from Fujian region showed a close relationship with SA00. Among the four Fujian ORFV strains, OV-GO was closest to OV-NP, and OV-YX was closest to OV-SJ1. Our analysis also revealed that eight ORFVs were more closely related to PCPV than to BPSV (Figure 4). The four Fujian ORFV strains shared 3–7 genes, and 4–9 genes with 85–95%, and < 85% amino acid identity with each other. Compared with SA00, the four Fujian ORFV strains shared 8–10 genes, and 5–11 genes with 85–95%, and < 85% amino acid identity. Compared with NA1/11, the four Fujian ORFV strains shared 8–10 genes and 5–11 genes with 85–95%, and < 85% amino acid identity. Compared with IA82, the four Fujian ORFV strains shared 23–28 genes, and 8–13 genes with 85–95%, and < 85% amino acid identity. Compared with NZ2, the four Fujian ORFV strains shared 23–26 genes, and 6–16 genes with 85–95%, and < 85% amino acid identity (Figure 5). From Figure 5, it is interesting to find that the four Fujian ORFV strains shared less than 10 genes with 85–95% amino acid identity with each other or with SA00, while they shared more than 20 genes with 85–95% amino acid identity with NA1/11, IA82, and NZ2. The result revealed ORFVs originating from goats are more similar to each other than to ORFVs originating from sheep. So we further analyzed single gene variations between goat ORFVs and sheep ORFVs. The phylogenetic trees based on the nucleotide sequences of each ORFV gene (132 in total) from this study and GenBank were constructed by the maximum-likelihood method using MEGA 5.0 software. Phylogenetic trees based on each of 47 genes (genes 007, 008, 009, 015, 018, 019, 020, 022, 023, 024, 028, 039, 041, 043, 044, 049, 050, 051, 054, 056, 057, 059, 060, 064, 068, 078, 079, 080, 081, 088, 091, 096, 106, 107, 111, 113, 114, 115, 117, 119, 120, 121, 122, 123, 125, 126, and 129) showed, with greater than 70% bootstrap support, that ORFVs with goat origination and sheep origination formed separate branches, suggesting great genetic variation presented in goat ORFVs and sheep ORFVs. Twelve of those 47 genes (genes 008, 015, 018, 041, 043, 050, 064, 079, 088, 117, 122, and 125) with bootstrap values greater than or equal to 99% at the node between goat branch and sheep branch and bootstrap values great than 50% at all nodes in goat branch and in sheep branch, are summarized in Figure 6, while the other 35 genes are summarized in Supplementary Figures S1–S3. The other genes (85 in total) lack phylogenetic signal to separate goat from sheep origins, and most of them can't form separate goat origination and sheep origination branches, while some genes (genes 010, 036, 037, 038, 061, 063, 065, 066, 082, 093, and 100) have phylogenetic signal to separate goat from sheep origins but with very low bootstraps (Supplementary Figures S4–S9). In order to further reveal genetic variation presented in goat ORFVs and sheep ORFVs, we analyzed the deduced amino acid sequences of gene 008, which can be easily distinguished as having originated from sheep or goats. From the phylogenetic tree based on the deduced amino acid sequences, the sequence analyses revealed that the four Fujian ORFVs shared a closer relationship to each other than to SA00, and NZ2 and IA82 shared a closer relationship than with NA1/11, whereas ORFVs with goat origination and sheep origination were sufficiently divergent (Figure 7A). Multiple alignment of amino acid sequences of gene 008 from eight ORFVs (five goat ORFVs and three sheep ORFVs) revealed 33 unique amino acid residues in goat originated ORFVs compared against sheep originated ORFVs (Figure 7B).

In order to investigate whether or not potential recombination events resulted in clustering discrepancies of the single gene trees, we performed genetic recombination analysis by using the Recombination Detection Program (RDP) (Martin and Rybicki, 2000). RDP, GENECONV, BootScan, MaxChi, Chimaera, SiScan and 3Seq methods with the default selection were used to perform the analyses in RDP4 (Heath et al., 2006). Results showed that no significant recombination breakpoints in all the genes in Figure 6 were identified, and only three genes (114, 121, and 126) listed in Supplementary Figures S2 and S3 in involved potential recombination event.

Forty-seven genes were identified that can be easily distinguished as having originated from sheep or goats (Figure 6, Supplementary Figures S1–S3). To test if codon selection occurs specifically in goat clade and sheep clade respectively, the branch site model A, which allows for ω > 1 along foreground branches, was compared with the null model A1, which only allows for ω ≤ 1 along foreground and back-ground branches. To identify amino acid sites potentially under positive selection, the parameter estimates from model were used. We used CODEML branch models in PAML (Yang, 2007) for an analysis of selection in goat branch and sheep branch of 47 genes respectively. Positive selections in the goat branch and sheep branch of gene 080, in the goat branch of gene 024 and 126 were identified (LRT *P* < 0.05). But only in goat branch of gene 080, a site (alignment position 100, corresponding to Pro-121 of GO, YX, SJ1, and SA00 strains and Pro-122 of NP strain) was identified under positive selection with posterior probability > 0.95. A potential site (alignment position 52, corresponding to Asn-124 of GO, YX, SJ1, NP, and SA00 strains) in goat branch of gene 024, was identified with posterior probability 0.940.

## Discussion

In our previous work, phylogenetic analysis based on ORFV011 and ORFV059 genes revealed that there was considerable heterogeneity among ORFV isolates from Fujian Province in China (Chi et al., 2013). Therefore, we sequenced and analyzed four more divergent ORFVs isolated from this region to investigate the heterogeneity of ORFVs based on the complete genome.

The genome structure of the four Fujian goat ORFV strains was the same as other ORFVs. The four Fujian goat ORFV genome length varied from 132,111 bp (OV-NP) to 139,886 bp (OV-GO) with 124–132 predicted genes. Like other poxviruses, all ORFV genomes contain a large central coding region bounded by two identical inverted terminal repeat (ITR) regions (Mercer et al., 1987; Fraser et al., 1990). All ORFVs analyzed in this study had about 64% G+C content. In all ORFVs except OV-NP, one gene (001/134) was completely located within the ITRs, while only 178 bp of gene 001 was located in the ITR of the OV-NP genome. Gene 134 in the OV-NP genome was disrupted due to a lack of initiation coding. Besides, after aligning the sequences external to the terminal *Bam*HI site among the genomes of ORFV strains, we found that OV-SJ1, OV-GO, and OV-YX contained these conserved telomere resolution sequences at both end of its genomes and OV-NP contained this sequence at the right end of the genome suggesting that the sequences of our four ORFVs strains are relative intact. The results from the aliment can be used to establish a generic resolution sequence for ORFV as 5′-T6 N8 TAAAT-3′, which is consistent with that of the vaccinia virus (Merchinsky, 1990).

The phylogenetic tree based on the complete genome showed the five goat ORFVs and four sheep ORFVs formed distinctly separate branches. Our four ORFV strains had close relations to SA00, indicating high heterogeneity among the four Fujian goat ORFVs. Analysis of the phylogenetic trees based on nucleotide sequences of each gene of ORFV, 47 genes in total were found to assist in easily distinguishing between goat and sheep originated ORFVs. Multiple alignment of the deduced amino acid sequence showed that gene 008 contained sufficient sequence heterogeneity for differentiating it as having sheep or goats as a host. ORFV of sheep or goat origin were comparatively analyzed at whole genome level in this study for the first and showed great variations, which provided an important theoretical basis for development host specificity vaccines to control orf.

The animal inoculation experiment showed that OV-GO had the strongest virulence, second was OV-YX, but OV-NP and OV-SJ1 showed low virulence. This result, taken together with the fact that genomic rearrangements have often been found to result in virus attenuation *in vivo* (Fleming et al., 1995; Rziha et al., 2000; McInnes et al., 2001), indicates that it is possible that deletion possibly attenuated the virulence in OV-NP and OV-SJ1. The function of genes 114–120 are unknown, except for gene 117 which is a GM-CSF inhibitory factor known as a virulence gene targeting the host's immune defense system (McInnes et al., 2005). It suggests the others are possibly involved in virulence, host range or pathogenesis.

## Author contributions

XC, XZ, SW, and SL participated in design of the study. XC, XZ, SL, WL, XH, YH, SW, DR, and ML performed the experiments. XZ, XC, SW, and SL analyzed the data and wrote the manuscript. All authors read and approved the final manuscript.

### Conflict of interest statement

The authors declare that the research was conducted in the absence of any commercial or financial relationships that could be construed as a potential conflict of interest.

## Supplementary material

Table S1 **ORFs of YX compared with the corresponding of ORFV strains GO, SJ1, NZ2 and SA00**.

Supplementary Figures S1–S3 **Phylogenetic analysis based on nucleotide sequence of ORFV single gene distinguishing goat and sheep origination**. These 35 genes can be easily distinguished as having originated from sheep or goats with greater than 70% bootstrap at the node between goat branch and sheep branch but bootstrap values less than 50% at the other nodes. The phylogenetic relationship was constructed by the maximum-likelihood method using MEGA 5.0 software. Numbers at the branching points indicate the bootstrap support calculated for 1000 replicates.

Supplementary Figures S4–S9 **Phylogenetic analysis based on nucleotide sequence of ORFV single gene unable distinguishing goat and sheep origination**. These 85 genes lack phylogenetic signal to separate goat from sheep origins or have phylogenetic signal to separate goat from sheep origins but with very low bootstraps. The phylogenetic relationship was constructed by the maximum-likelihood method using MEGA 5.0 software. Numbers at the branching points indicate the bootstrap support calculated for 1000 replicates.
