# Supplementary figures and images for: Genome analysis of orf virus isolates from goats in the Fujian Province of southern China
Source: Front Microbiol. 2015 Oct 23;6:1135. doi: 10.3389/fmicb.2015.01135 (PMC4616995; doi:10.3389/fmicb.2015.01135)

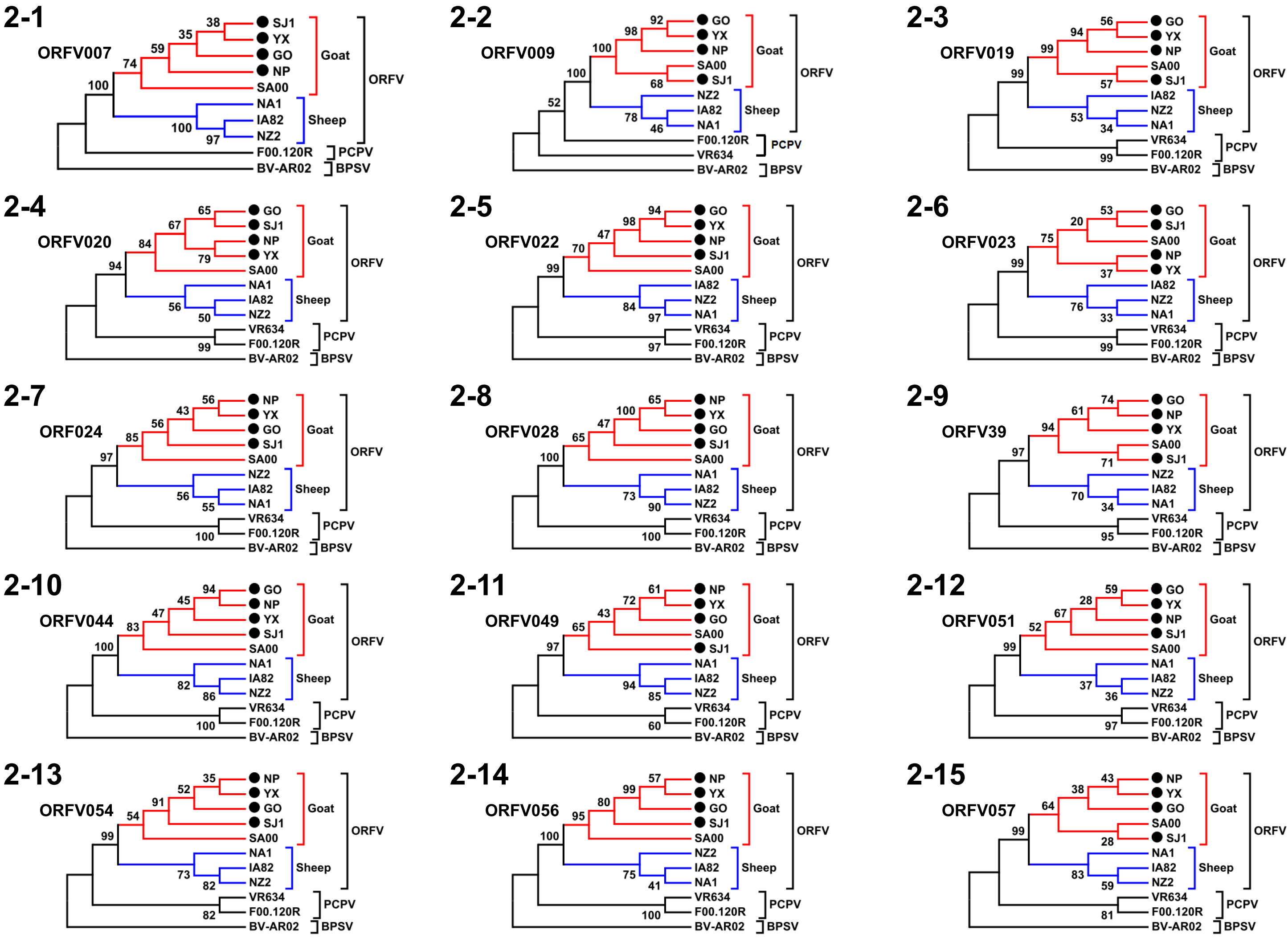

Supplement: Supplementary Figures S1–S3 — Phylogenetic analysis based on nucleotide sequence of ORFV single gene distinguishing goat and sheep origination. These 35 genes can be easily distinguished as having originated from sheep or goats with greater than 70% bootstrap at the node between goat branch and sheep branch but bootstrap values less than 50% at the other nodes. The phylogenetic relationship was constructed by the maximum-likelihood method using MEGA 5.0 software. Numbers at the branching points indicate the bootstrap support calculated for 1000 replicates. [file FigureS1.TIF]

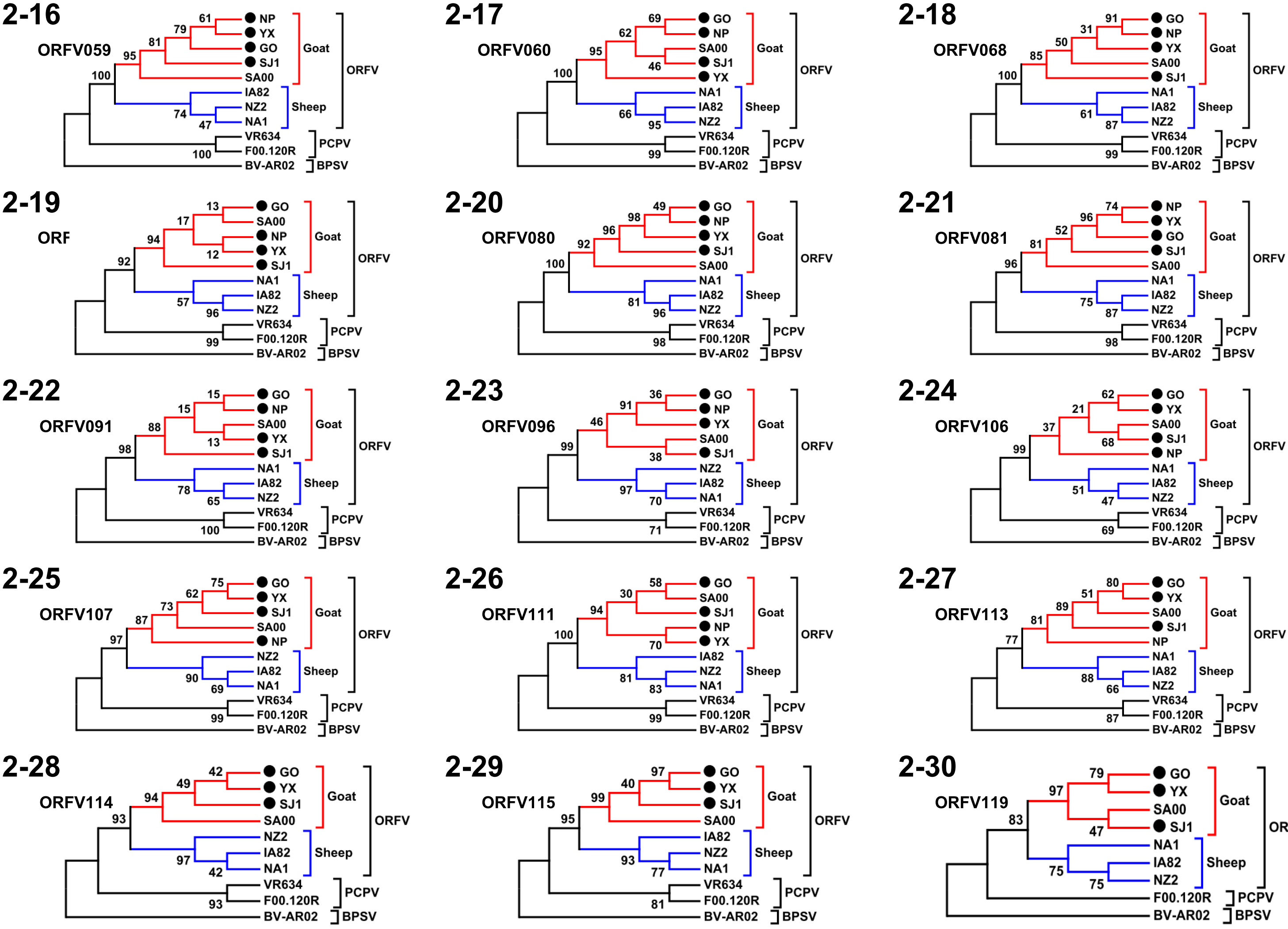

Supplement: Supplementary file 3 [file FigureS2.TIF]

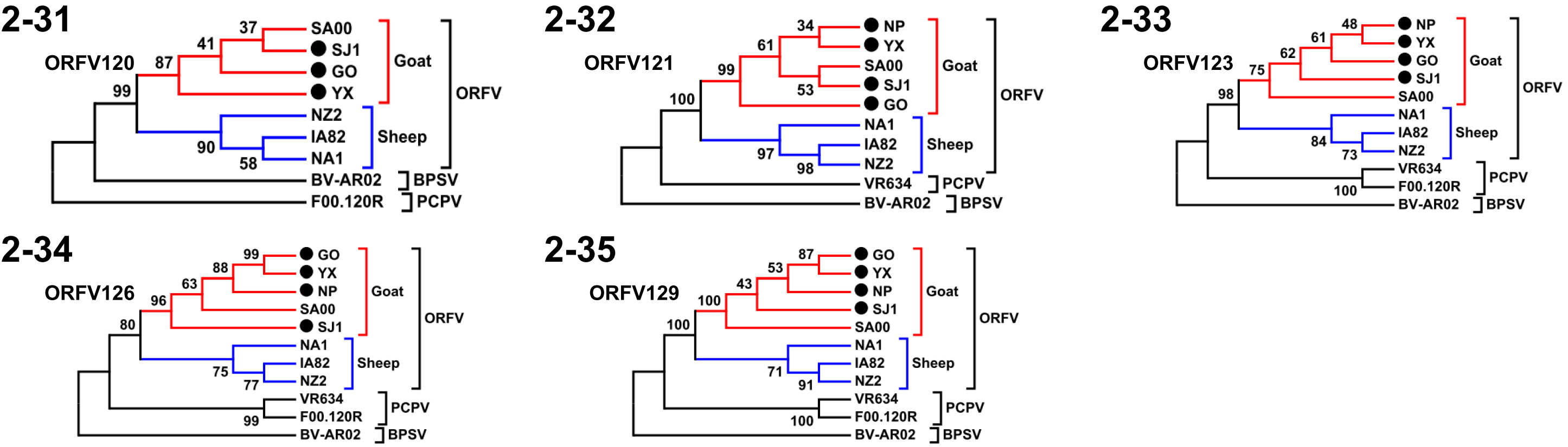

Supplement: Supplementary file 4 [file FigureS3.TIF]

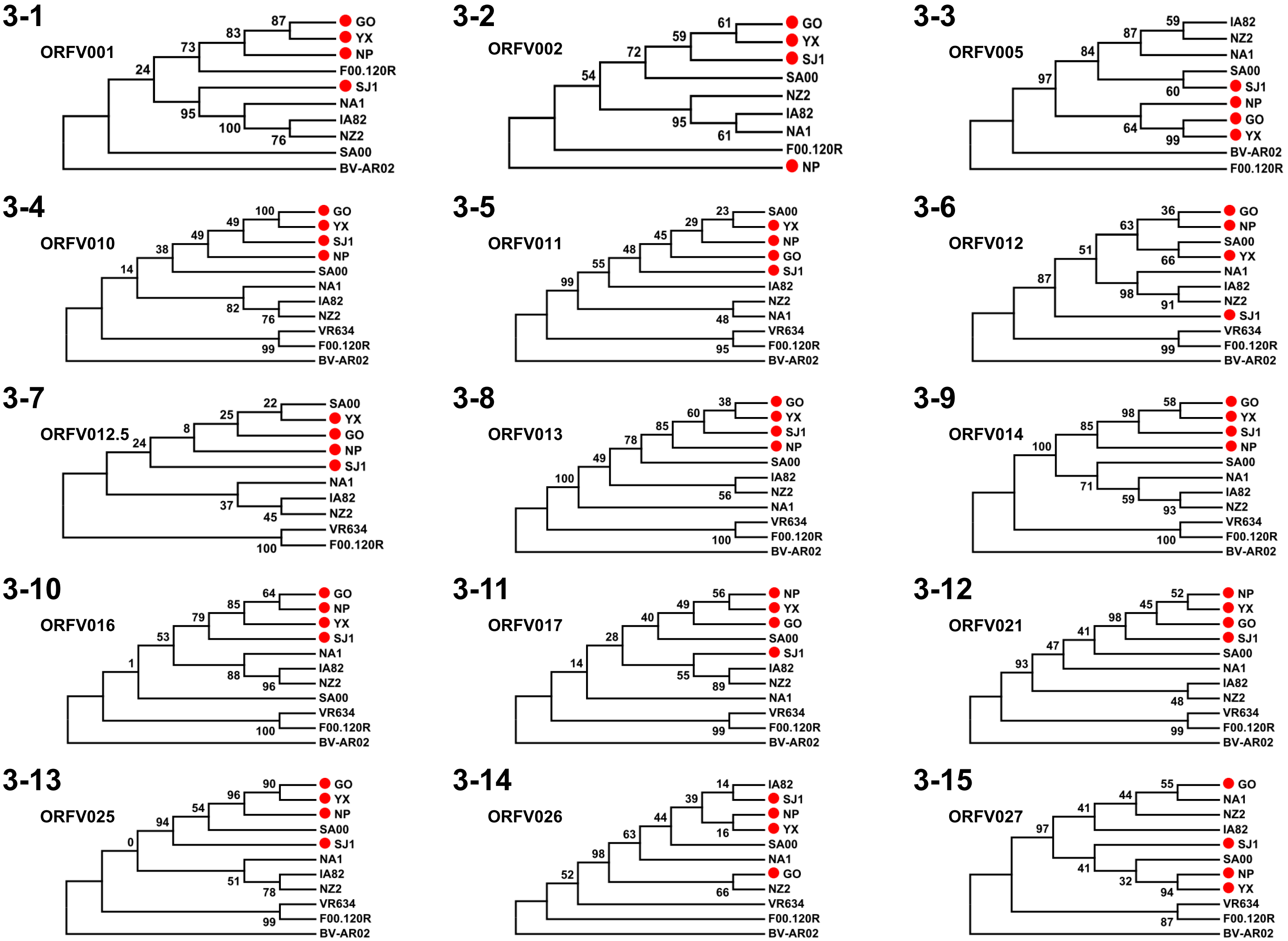

Supplement: Supplementary Figures S4–S9 — Phylogenetic analysis based on nucleotide sequence of ORFV single gene unable distinguishing goat and sheep origination. These 85 genes lack phylogenetic signal to separate goat from sheep origins or have phylogenetic signal to separate goat from sheep origins but with very low bootstraps. The phylogenetic relationship was constructed by the maximum-likelihood method using MEGA 5.0 software. Numbers at the branching points indicate the bootstrap support calculated for 1000 replicates. [file FigureS4.TIF]

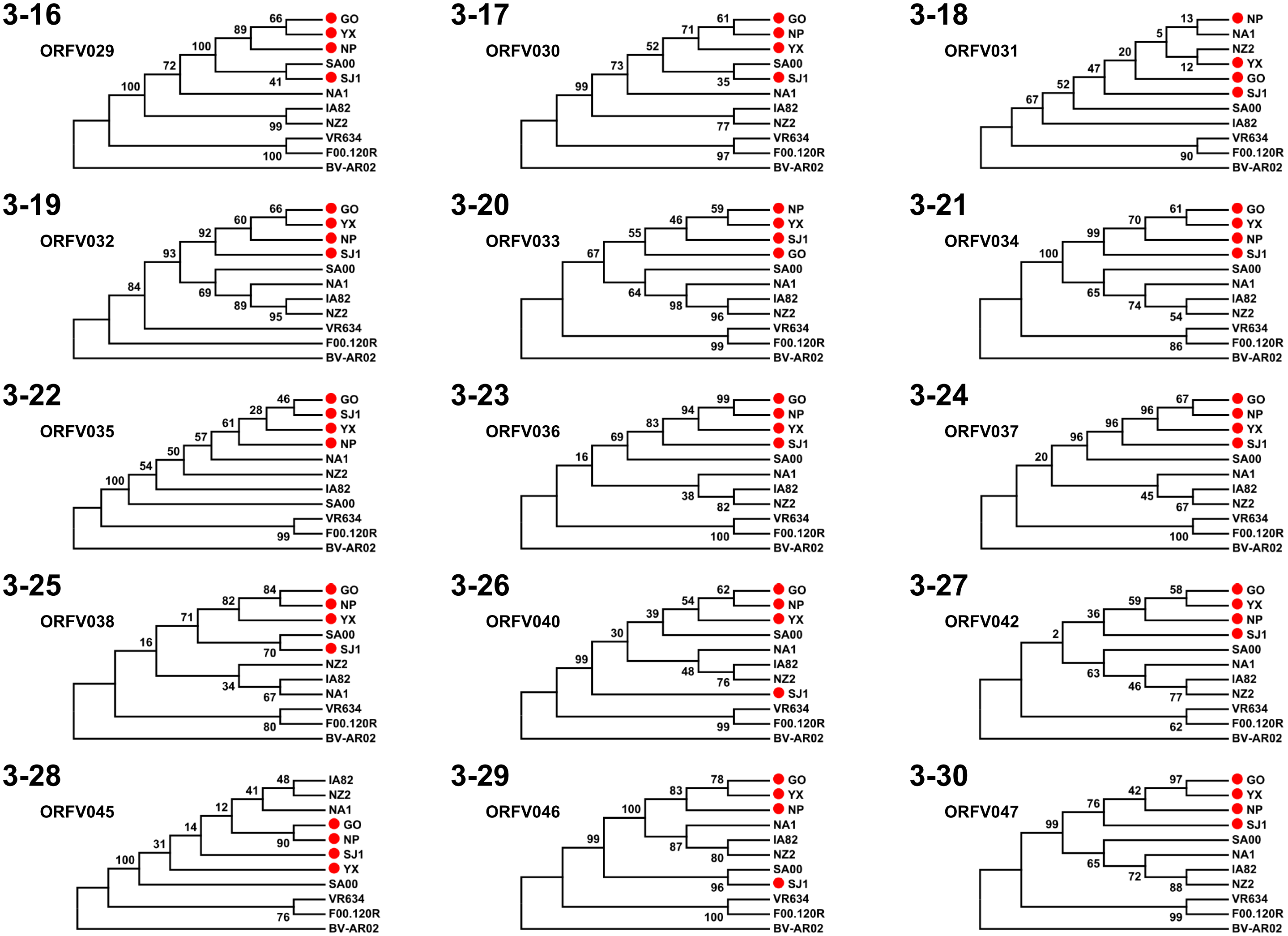

Supplement: Supplementary file 6 [file FigureS5.TIF]

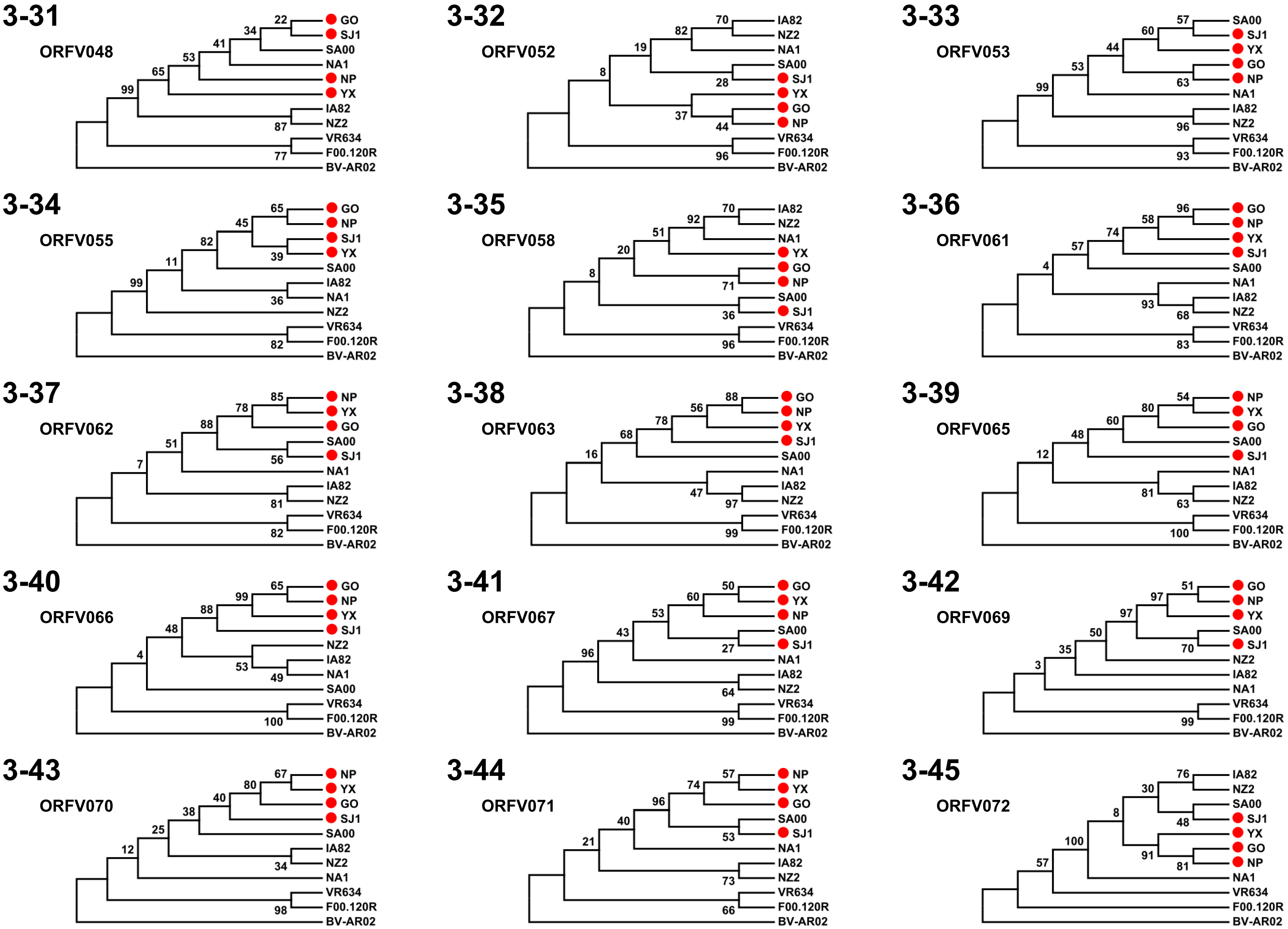

Supplement: Supplementary file 7 [file FigureS6.TIF]

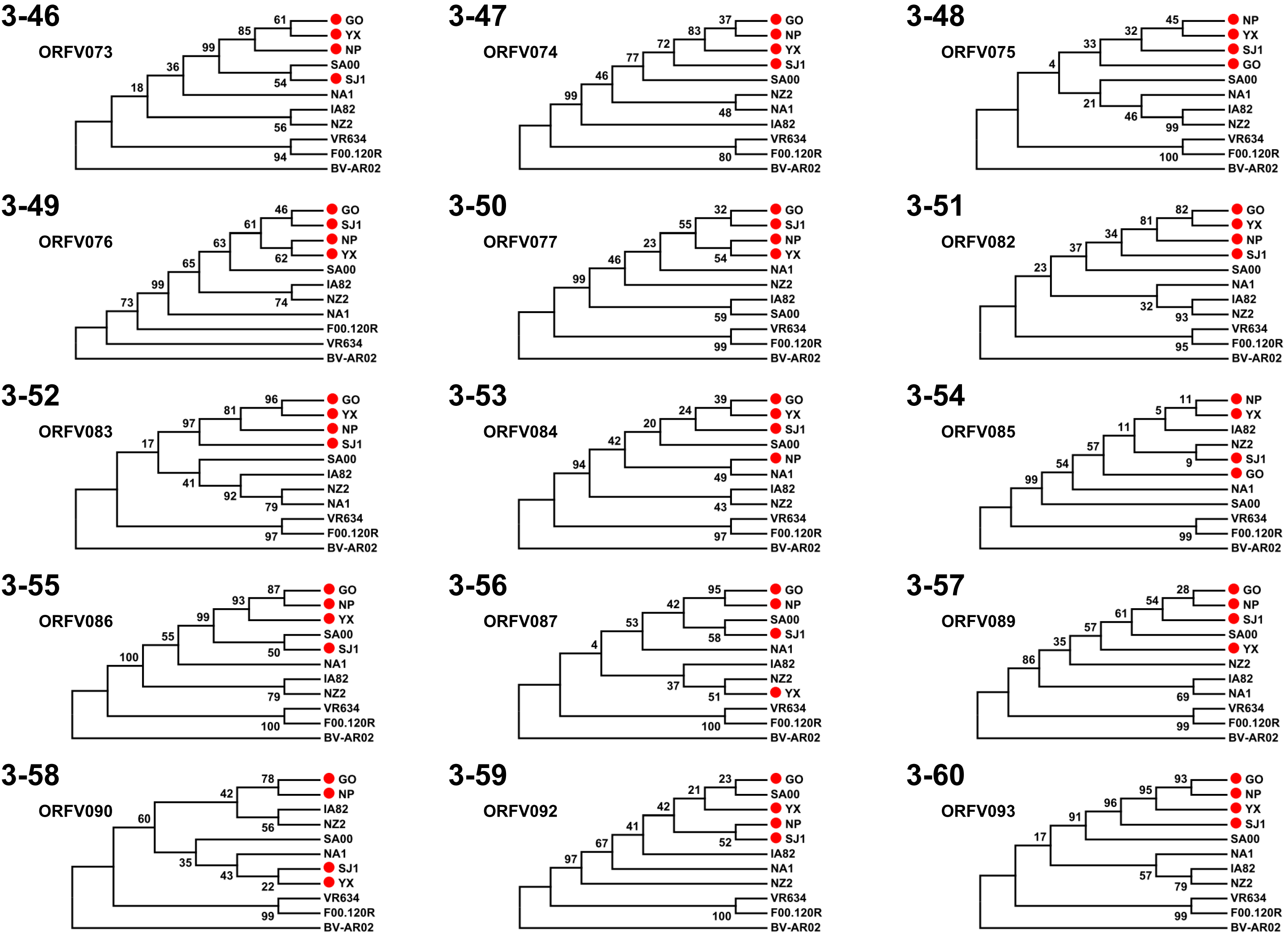

Supplement: Supplementary file 8 [file FigureS7.TIF]

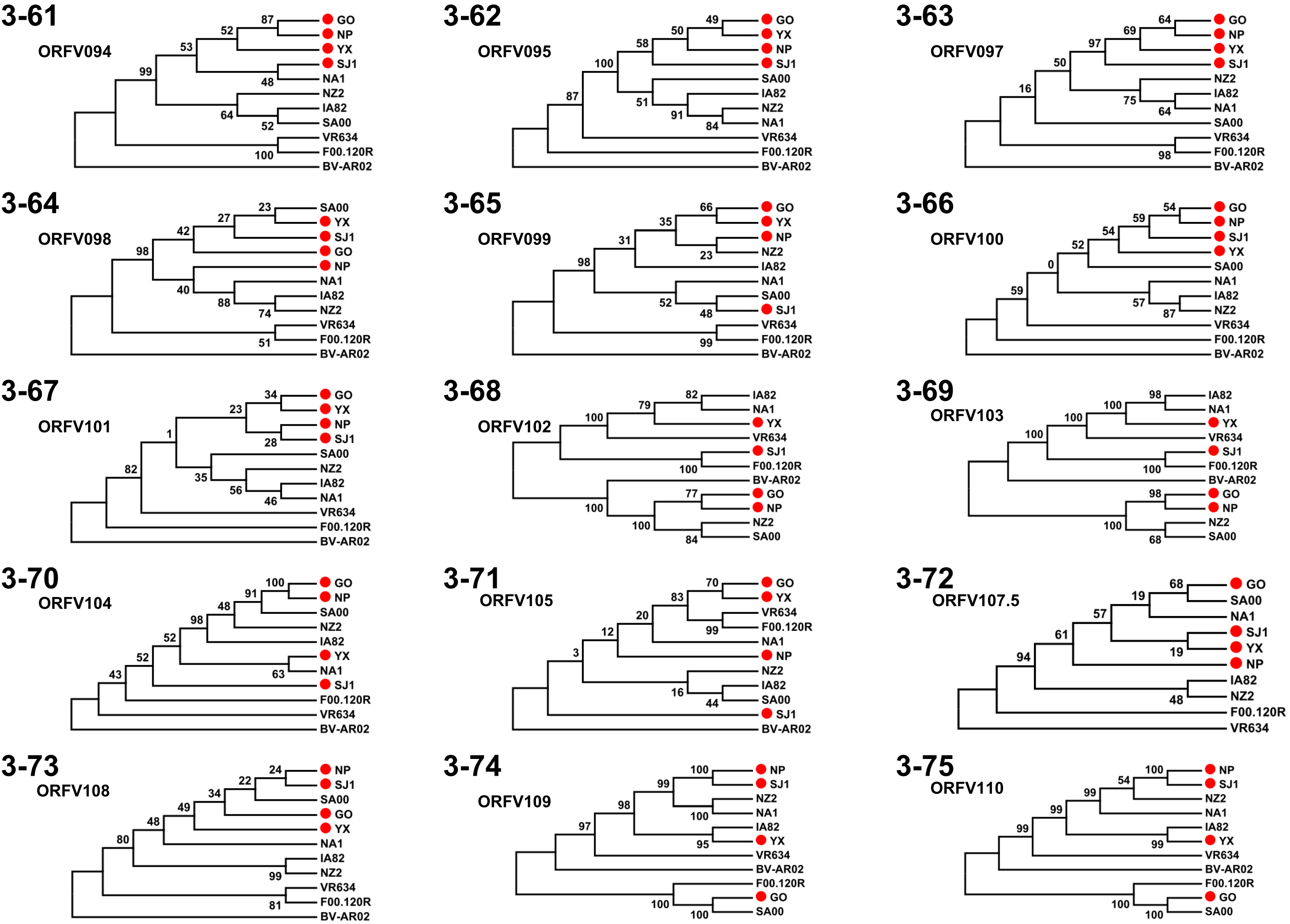

Supplement: Supplementary file 9 [file FigureS8.TIF]

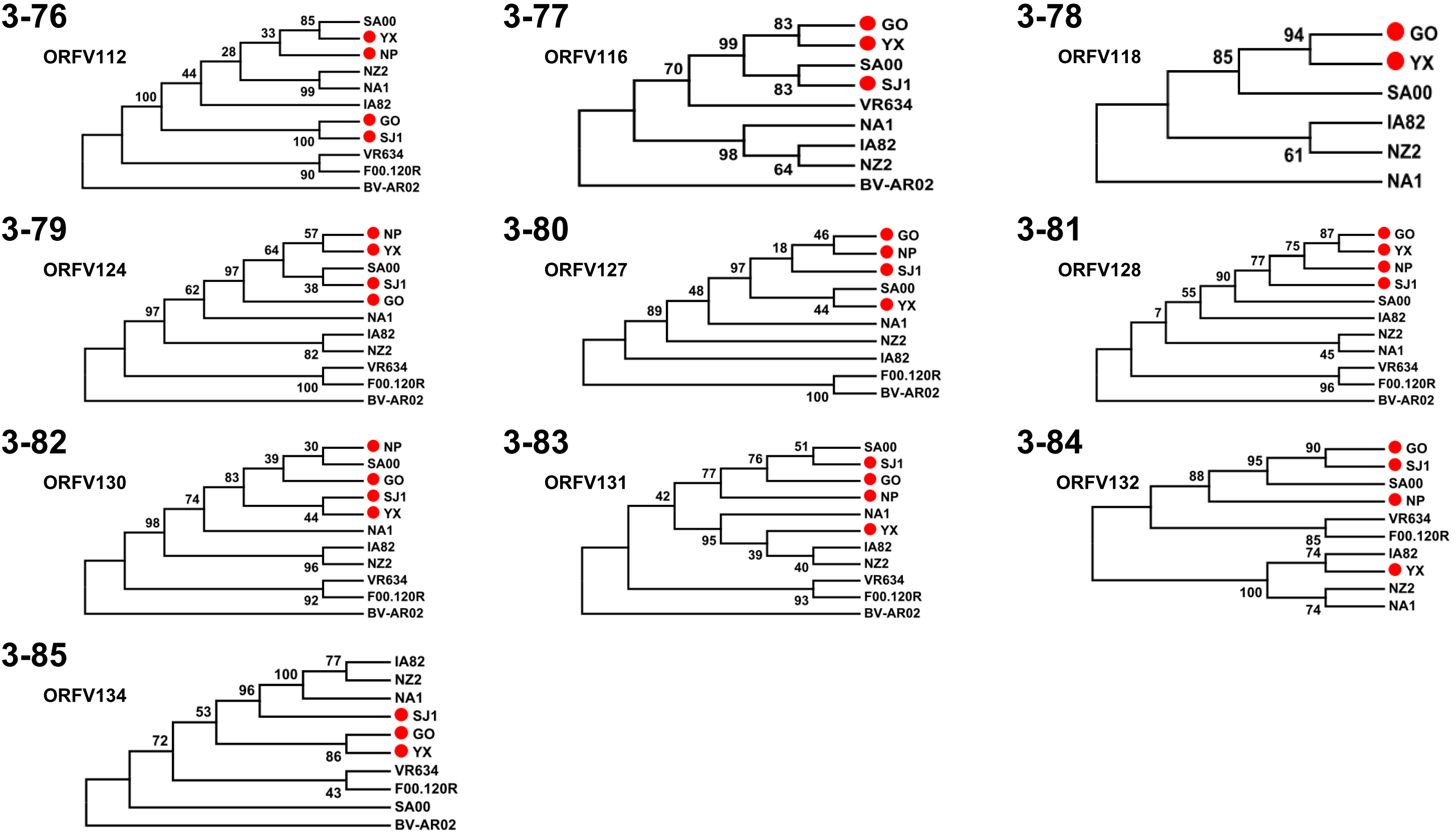

Supplement: Supplementary file 10 [file FigureS9.TIF]
